# Primary Small Intestinal Volvulus in a 91-Year-Old Patient: A Rare Case of Successful Laparotomy With Resection and Anastomosis

**DOI:** 10.7759/cureus.103804

**Published:** 2026-02-17

**Authors:** Yasmeen S Rahmi, Nisreen M Awad, Rama N Alshanawani, Ibrahim Elnogoomi

**Affiliations:** 1 General Surgery, College of Medicine, University of Sharjah, Sharjah, ARE; 2 General Surgery, Kuwait Hospital Sharjah, Sharjah, ARE

**Keywords:** acute small bowel obstruction, case report, intestinal resection and anastomosis, jejunal diverticulosis, primary small intestinal volvulus

## Abstract

Primary small intestinal volvulus (PSIV) is a rare cause of acute bowel obstruction in adults and is exceptionally uncommon in the very elderly. It occurs when the small intestine twists around its mesenteric axis without an identifiable secondary cause, such as adhesions or hernias. This case report describes a 91-year-old woman who presented with abdominal pain and minimal rectal bleeding, posing a diagnostic challenge in the context of multiple comorbidities and atypical symptoms. CT imaging demonstrated a persistent “whirlpool sign,” raising suspicion for volvulus, and the patient underwent emergency laparotomy with detorsion and resection of nonviable segments. Despite advanced age and high perioperative risk, the patient achieved a favorable postoperative outcome following timely surgical intervention. This case highlights the diagnostic difficulty of PSIV in the very elderly population and underscores the importance of early operative management to prevent progression to bowel ischemia and reduce morbidity and mortality.

## Introduction

Primary small intestinal volvulus (PSIV) is an uncommon cause of acute intestinal obstruction, especially in adults, and is rarely documented in the elderly population [1]. It is caused by twisting of the small bowel around its mesenteric axis without an identifiable secondary cause, such as adhesions or tumors. The clinical presentation is often nonspecific, typically including acute abdominal pain, nausea, vomiting, abdominal distension, and obstipation, which can mimic other causes of small bowel obstruction and contribute to delayed diagnosis [2]. Elderly patients may present with more subtle symptoms, leading to further diagnostic uncertainty [1].

Initial imaging commonly begins with a plain abdominal radiograph, which may demonstrate dilated small bowel loops and air-fluid levels but lacks diagnostic specificity. CT of the abdomen is the preferred preoperative imaging modality, as it can identify characteristic findings such as the whirl or whirlpool sign, closed-loop obstruction, and features suggestive of bowel ischemia [3]. Despite these imaging advances, preoperative diagnosis remains challenging, and definitive diagnosis is frequently established intraoperatively [2,3].

We present the case of a 91-year-old patient with PSIV who underwent emergency laparotomy with segmental resection and primary anastomosis, resulting in a favorable postoperative outcome. This case highlights the importance of maintaining a high index of suspicion for this rare condition, even in advanced age, and the potential for successful recovery with timely surgical management.

## Case presentation

A 91-year-old Emirati woman, bedridden with multiple chronic illnesses, was brought to the emergency department by the National Ambulance in the evening with new-onset abdominal pain and a single episode of fresh blood per rectum. According to her caregivers, the bleeding was minimal, appearing as light red streaks mixed with stool. There was no associated vomiting, diarrhea, fever, jaundice, hematuria, or chest pain.

The patient had been discharged recently from Kuwaiti Hospital in Sharjah, where she was admitted for management of common bile duct (CBD) and gallbladder stones. She was scheduled for an elective endoscopic retrograde cholangiopancreatography (ERCP) on the same day as the presentation. Aspirin, which she had been taking for ischemic heart disease, was stopped four days prior to admission.

She had a significant past medical history, including cerebrovascular accident with residual immobility; chronic obstructive pulmonary disease with bronchiectasis; ischemic heart disease with left ventricular dysfunction; mild pulmonary hypertension; dementia; iron deficiency anemia; and recurrent urinary tract infections and pneumonia. Surgical history included percutaneous endoscopic gastrostomy tube insertion in 2023 (later removed) and dilation and curettage for pyometra in 2024. There was no known family history of GI or bleeding disorders. She reported no allergies and had no history of tobacco, alcohol, or recreational drug use.

On arrival, the patient appeared cachectic and mildly uncomfortable but afebrile. She was alert, though communication was limited. Vital signs showed a temperature of 36.2 °C (tympanic), a heart rate of 65 bpm, a respiratory rate of 19 breaths/min, a blood pressure of 145/67 mmHg, and an oxygen saturation of 98% on room air. She weighed approximately 55 kg (BMI 19.7).

Physical examination revealed a soft but mildly distended abdomen with generalized mild tenderness, without guarding or rebound. A nontender, nonreducible swelling was noted in the left inguinal region. Digital rectal examination confirmed the presence of a small amount of fresh blood mixed with stool, without palpable masses. Chest auscultation was clear, with no added sounds, and cardiac examination revealed normal heart sounds without murmurs. Neurologically, she scored 15 on the Glasgow Coma Scale, with no new focal deficits.

Initial laboratory results, shown in Table 1, demonstrated anemia with a hemoglobin level of 8.9 g/dL. The white blood cell count was 3.03 × 10³/µL, with marked neutrophilia (81.7%). Inflammatory markers were elevated, with CRP at 116 mg/L and D-dimer at 2.4 mg/L. Venous blood gas analysis demonstrated mild metabolic acidosis with compensatory respiratory changes. Electrolyte and renal studies revealed sodium 137 mmol/L, potassium 2.64 mmol/L, chloride 100 mmol/L, creatinine 63 µmol/L, and an elevated anion gap of 16. The glucose level was 7.5 mmol/L, and the estimated glomerular filtration rate was 80 mL/min/1.73 m². Liver function tests, performed in the context of suspected CBD stones, were within normal limits.

**Table 1:** Laboratory investigations on admission

Parameter	Value	Reference range
White blood count (×10³/µL)	3.03 (Low)	4.0-11.0 × 10³/µL
Red blood cells (×10⁶/µL)	3.3 (Low)	3.8-5.1 × 10⁶/µL
Hemoglobin (g/dL)	8.9 (Low)	12.0-15.5 g/dL
Hematocrit (%)	27.6 (Low)	36-46%
Mean corpuscular volume (fL)	82.9	80-100 fL
Mean corpuscular hemoglobin (pg)	26.9 (Low)	27-33 pg
Mean corpuscular hemoglobin concentration (g/dL)	32.2	32-36 g/dL
Red cell distribution width (%)	15 (High)	11.5-14.5%
Platelets (×10³/µL)	258	150-400 × 10³/µL
Neutrophils (%)	81.7 (High)	40-75%
Lymphocytes (%)	12.5 (Low)	20-45%
CRP (mg/L)	116 (High)	<5 mg/L
D-dimer (mg/L FEU)	2.4 (High)	<0.5 mg/L FEU
Venous pH	7.316	7.31-7.41
pCO₂ (mmHg)	36.1 (Low)	41-51 mmHg
pO₂ (mmHg)	54.6 (High)	30-50 mmHg
HCO₃⁻ (mmol/L)	18 (Low)	22-26 mmol/L
Lactate (mmol/L)	1.9 (High)	0.5-1.6 mmol/L
Sodium (mmol/L)	137	135-145 mmol/L
Potassium (mmol/L)	2.64 (Low)	3.5-5.0 mmol/L
Chloride (mmol/L)	100	98-106 mmol/L
Random glucose (mmol/L)	7.5	3.9-7.8 mmol/L
Urea/blood urea nitrogen (mmol/L)	5.4	2.5-7.8 mmol/L
Creatinine (µmol/L)	63	45-90 µmol/L
Estimated glomerular filtration rate (mL/min/1.73 m²)	80	≥90 mL/min/1.73 m²

Given her presentation of abdominal discomfort and inguinal swelling, the possibility of intra-abdominal pathology, such as an internal hernia or femoral hernia, was considered. The patient was admitted to the female surgical ward for further evaluation. Management included keeping her nil per os (NPO), insertion of a nasogastric tube, urinary catheter placement for fluid balance monitoring, IV fluids, preoperative laboratory investigations, and crossmatching of two units of blood. She was prepared for potential diagnostic laparoscopy or laparotomy, depending on operative findings, while her planned ERCP remained on the schedule. One dose of IV ceftriaxone 2 g and one dose of IV paracetamol 1 g were administered, and close monitoring of vital signs was initiated.

**Figure 1:**
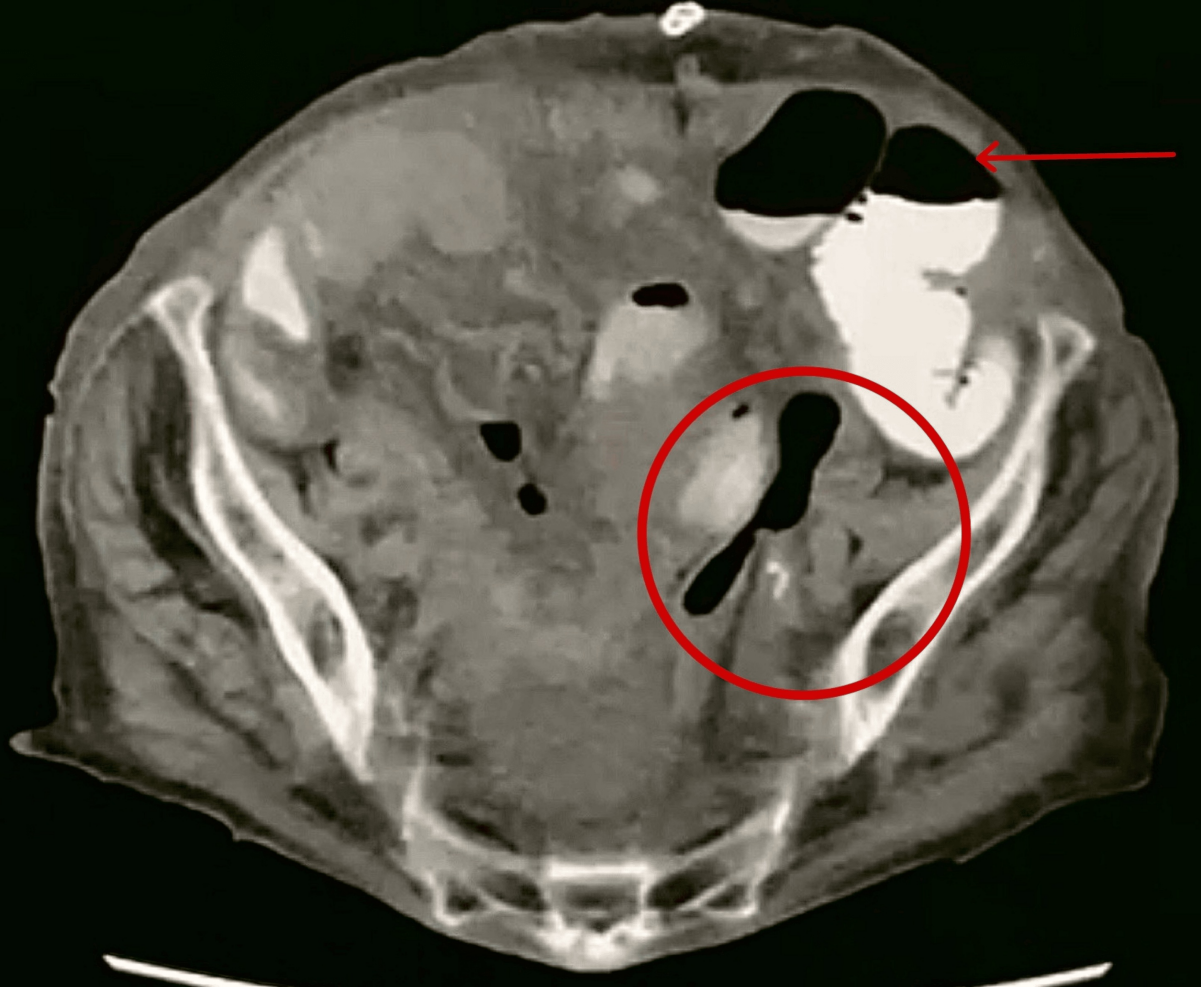
Axial CT with oral contrast demonstrating air-fluid levels (arrow) and incomplete passage of contrast to the distal bowel (circle)

**Figure 2:**
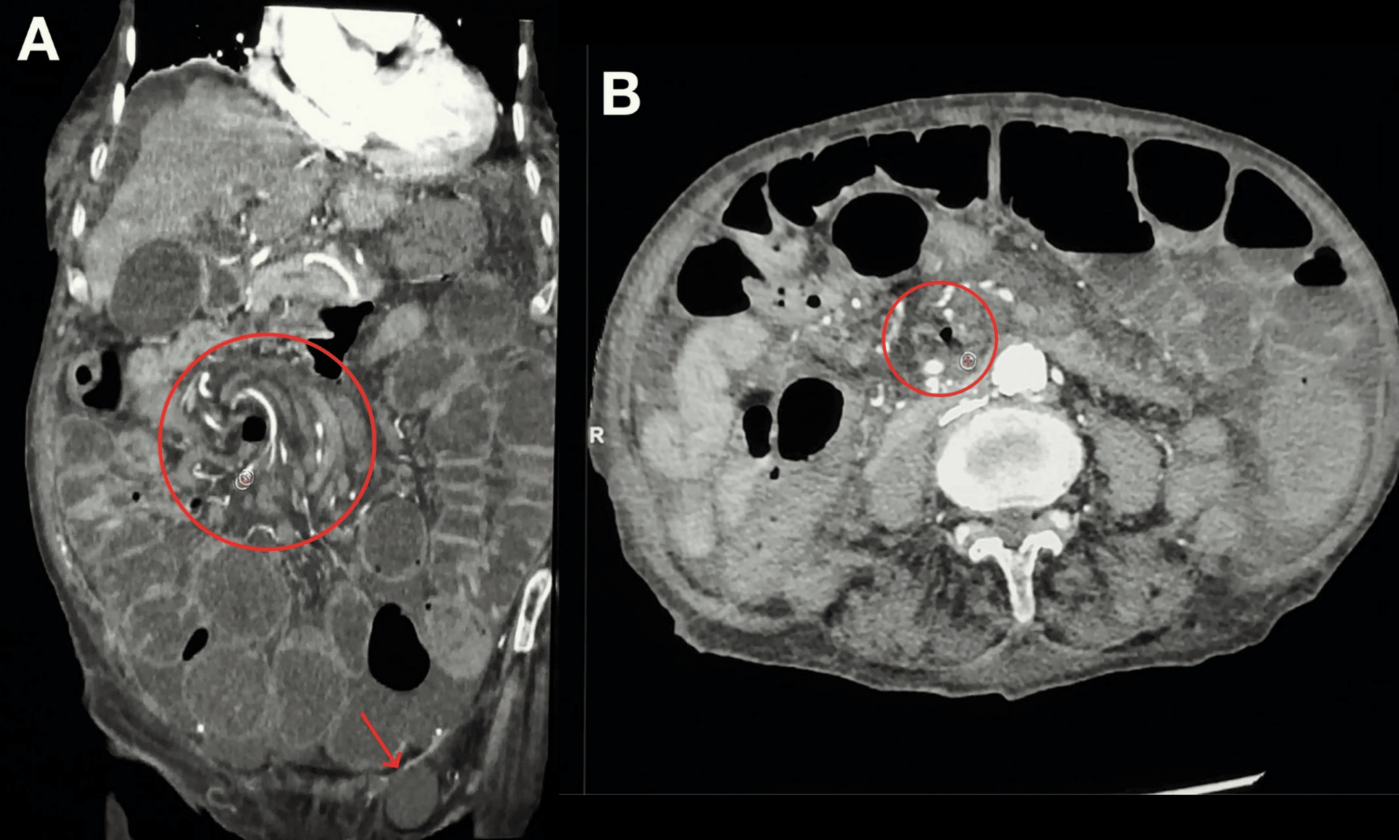
Contrast-enhanced CT images demonstrating the whirlpool sign (A) Coronal CT image demonstrating the whirlpool sign (circle) with an associated small left femoral hernia (arrow). (B) Axial contrast-enhanced CT image demonstrating the whirlpool sign (circle).

After hemodynamic stabilization, a noncontrast CT abdomen was performed and showed dilated proximal small bowel loops with collapsed distal ileal segments, consistent with a subacute mechanical small bowel obstruction. Oral contrast failed to reach the distal bowel, suggesting a tight distal obstruction (Figure 1). A persistent whirlpool sign in the right paramedian abdomen (Figure 2), representing twisting of the mesenteric vessels and bowel around a fixed point, raised concern for volvulus due to an adhesion band or internal hernia. Additional findings included mild to moderate ascites within the peritoneal cavity, a dilated CBD with distal stones, intrahepatic biliary radicle dilatation (IHBRD), and a left adnexal cystic lesion, likely of ovarian origin.

A small left femoral hernia containing cystic content (Figure 2A) and a small sliding hiatal hernia were also noted, both unchanged from prior imaging.

Overall, the CT findings suggested a persistent subacute small bowel obstruction with an unchanged whirlpool sign shown in Figure 2 and failure of oral contrast to progress distally, raising a strong suspicion for a tight mechanical obstruction, possibly due to volvulus (Figure 1). Persistent CBD dilatation with distal stones and IHBRD remained evident, along with ascites, a left adnexal cyst, and small femoral and hiatal hernias, all unchanged from previous studies.

The patient was taken for exploratory laparotomy under general anesthesia. She was positioned supine, and the surgical field was sterilized using 2% chlorhexidine and 70% isopropyl alcohol. A midline abdominal incision was made along the linea alba, extending from the xiphoid process to just below the umbilicus. The incision was carried through the subcutaneous tissue and linea alba using electrocautery, providing an avascular entry into the peritoneal cavity.

**Figure 3:**
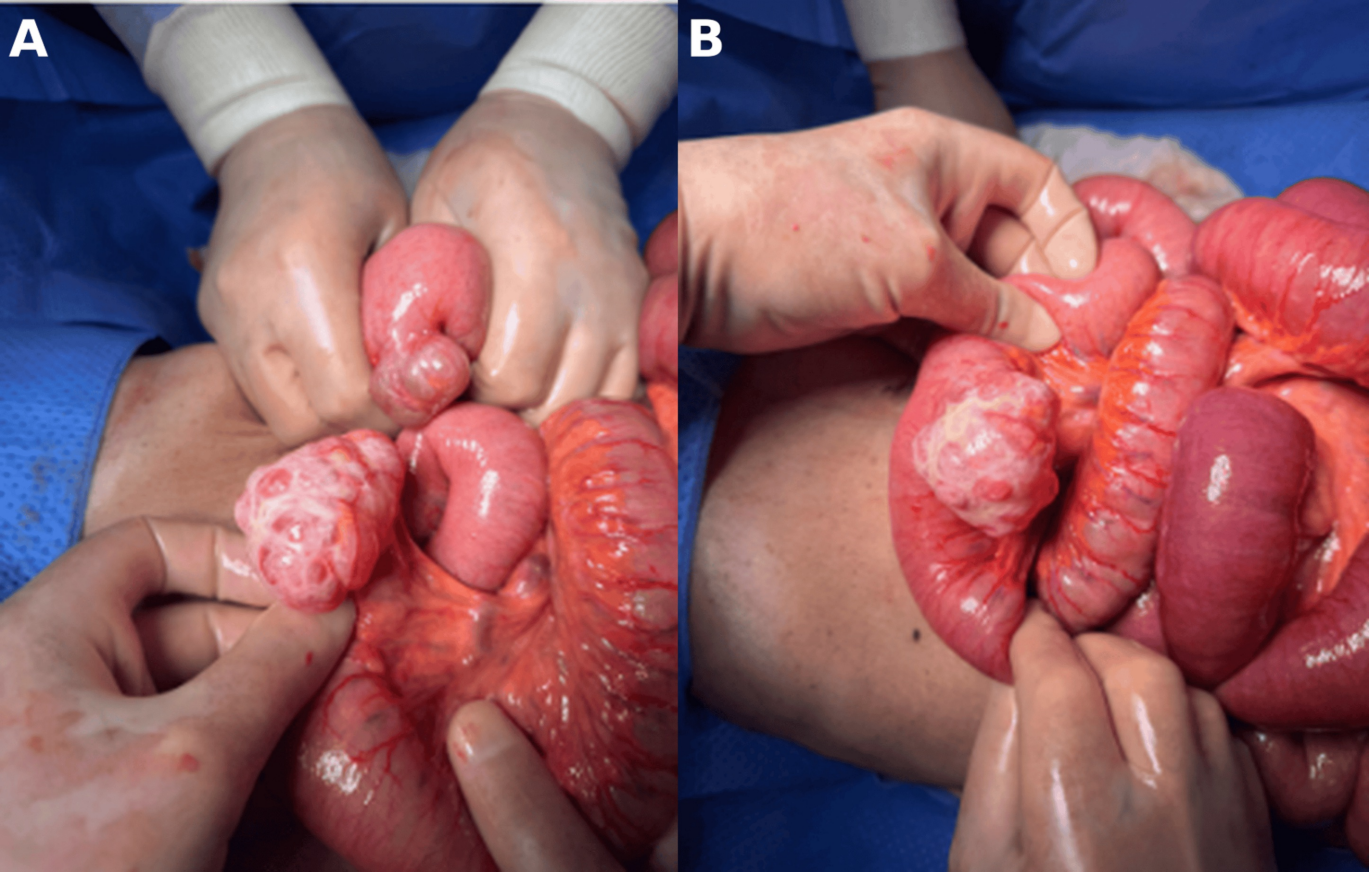
Intraoperative findings (A) Two thin-walled small-bowel diverticula on the antimesenteric border: the larger diverticulum has a broad base with a translucent serosal covering, while the smaller diverticulum is located proximally with similar morphology. (B) A clearer intraoperative view of the larger thin-walled diverticulum on the antimesenteric border of the small bowel.

**Figure 4:**
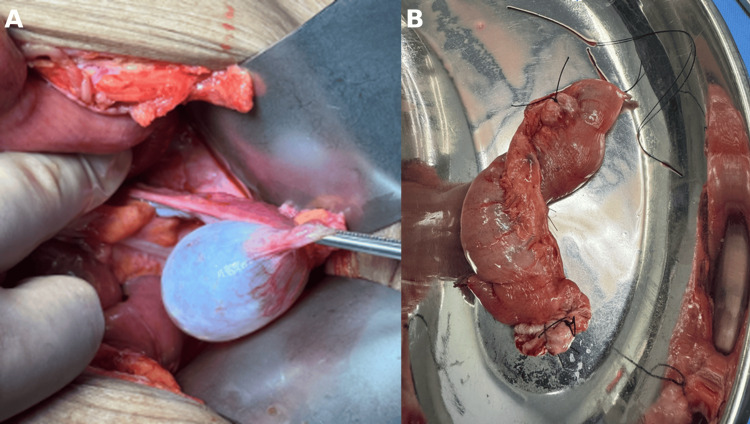
Intraoperative findings (A) Incidental right ovarian cyst with a smooth, translucent surface and well-defined borders without evidence of torsion or rupture. (B) Resected segment of small bowel containing the diverticula, sent for histopathology.

Upon entering the cavity, serous free fluid began leaking out, indicating congestion, impending ischemia, and chronic obstruction. Complete inspection of the small and large bowels revealed that the entire small bowel was twisted around its long, narrow mesentery, consistent with a primary volvulus of the small intestine. Immediate detorsion was performed. Further exploration from the duodenojejunal (DJ) junction to the ileocecal valve showed no abnormalities except for two diverticula located on the antimesenteric border: one large diverticulum with a wide base located 50 cm from the DJ junction and a smaller one located 40 cm from the DJ junction (Figure 3). These thin-walled diverticula were resected, and a primary anastomosis was performed using a linear stapler. Further exploration of the abdomen revealed an incidental finding of a right ovarian cyst (Figure 4A), which was then drained and marsupialized.

The bowel was then checked for proper alignment, and irrigation with normal saline followed by suction was performed. Hemostasis was secured, and the fascia was closed using Stratafix size 1. Three subcutaneous sutures with 3-0 Vicryl were placed to secure the umbilicus. The skin was closed with a skin stapler, and an antiseptic dressing was applied.

There were no intraoperative complications, and the patient tolerated the procedure well. The resected diverticula were sent for histopathological analysis (Figure 4B), and the peritoneal fluid was sent for culture.

Postoperatively, the patient was admitted to the ICU for close monitoring. She was maintained on NPO status and started on parenteral nutrition tailored to her weight and nutritional requirements. She remained free of nausea or vomiting. She experienced early postoperative hypotension, which responded to IV fluid resuscitation, and electrolyte abnormalities, including hypomagnesemia and hypokalemia, were corrected during her ICU stay.

She developed leukocytosis with rising CRP, for which empirical antibiotics were initiated. Peritoneal fluid culture later grew *Staphylococcus haemolyticus*, prompting adjustment of antimicrobial therapy according to sensitivities. A postoperative CT scan showed no complications, and oral contrast administered via the nasogastric tube passed smoothly through the anastomosis without evidence of leak or obstruction.

**Figure 5:**
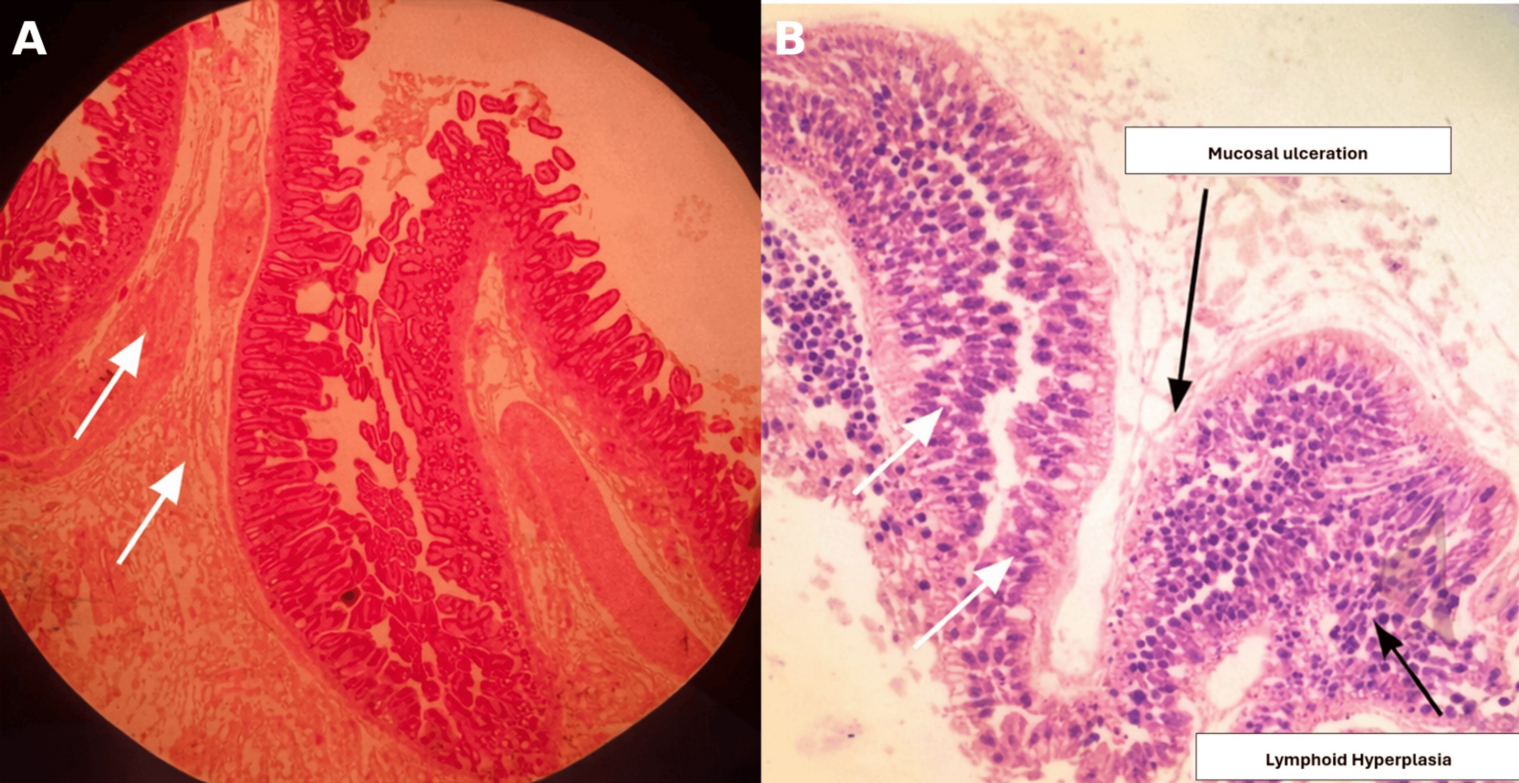
Histopathological examination of resected jejunal diverticulum (H&E stain) (A) Low-power view showing small intestinal mucosa with partial villous atrophy and architectural distortion, with underlying chronic inflammatory changes. (B) Higher-power view demonstrating mucosal ulceration, lymphoid hyperplasia, and chronic inflammatory infiltrates. No evidence of granulomas or malignancy is identified.

Histopathological examination (Figure 5) of the resected jejunal diverticula revealed partial villous atrophy, mucosal ulceration, lymphoid hyperplasia, and chronic inflammatory changes, without granulomas or malignancy, confirming jejunal diverticulosis.

On postoperative day 5, a clear liquid diet was initiated and well tolerated. She was transferred from the ICU to the general surgical ward on day 6 for continued observation. Her recovery progressed steadily, and she was discharged on day 22 with proton pump inhibitor therapy, ferrous sulfate (for iron deficiency anemia), a multivitamin, and lactulose 25 mL daily. The family received counseling regarding the importance of adequate nutrition and assisted feeding at home. Her usual home medications were resumed.

At her two-week outpatient follow-up, the patient appeared clinically stable, with a well-healed wound and no GI complaints. She was also scheduled for the ERCP that had been planned prior to surgery but was deferred and subsequently arranged at this visit.

## Discussion

PSIV is a rare cause of intestinal obstruction in adults and is exceptionally uncommon in the very elderly [1,2]. In contrast, secondary volvulus, most often related to adhesions, tumors, or hernias, is far more prevalent in this age group [3]. PSIV is defined by the absence of an underlying structural lesion, and its pathogenesis remains incompletely understood. Current literature suggests several contributing mechanisms, including sudden hyperperistalsis after prolonged fasting followed by a large meal and congenital mesenteric variations, such as an abnormally elongated mesentery, which may persist into older age and predispose the bowel to torsion [1,2,4].

PSIV has been reported more frequently in regions with higher dietary fiber intake, including parts of Africa, the Middle East, and Asia [2,4]. In elderly patients, diagnosis can be particularly challenging due to atypical presentations, the presence of multiple comorbidities, and the limited ability of imaging studies to reliably exclude bowel ischemia [1].

Our patient, a 91-year-old woman, represents one of the oldest reported cases of PSIV [5]. Most previously published series describe patients in their third to sixth decades of life, with only isolated reports in the very elderly [2,5]. Age-related bowel changes, mesenteric laxity, and comorbid cardiovascular disease may increase the risk of poor outcomes in this population [3,6]. Nevertheless, timely surgical intervention remains the cornerstone of management, as delay in operative treatment is associated with progression to bowel gangrene, sepsis, and mortality rates exceeding 40% [1,3].

In this case, the patient’s laboratory profile demonstrated leukopenia followed by postoperative leukocytosis and markedly elevated inflammatory markers (CRP 116 mg/L and procalcitonin 6.63 ng/mL), reflecting both the systemic inflammatory response and the physiological stress of surgery. Her preoperative venous blood gas analysis showed metabolic acidosis with elevated lactate, a finding consistent with tissue hypoperfusion and raising suspicion for evolving bowel ischemia [3]. These results highlight the importance of integrating laboratory and imaging findings with clinical judgment in elderly patients, whose symptoms may be muted by baseline frailty or dementia [1,3].

Radiologically, the non-contrast CT scan revealed a persistent “whirlpool sign,” described as the most characteristic imaging finding of volvulus, along with proximal small bowel dilatation and collapsed distal ileum. However, definitive diagnosis usually requires operative exploration, as CT sensitivity is not absolute [1]. In this patient, the imaging findings, together with inguinal swelling and obstructive symptoms, prompted early surgical referral despite her high anesthetic risk [3].

Current literature consistently supports early surgical intervention to prevent progression to ischemia and necrosis. Detorsion alone may be attempted if the bowel is viable, but segmental resection with primary anastomosis is indicated when nonviable segments are found and the patient’s physiology allows it [1,3]. In elderly patients with multiple comorbidities, conservative approaches or creation of a stoma are sometimes favored to reduce operative stress [6]. However, in this case, laparotomy with resection and anastomosis was performed successfully despite the patient’s advanced age, in accordance with best practice [1,3].

Reported postoperative complications include ileus, anastomotic leak, intra-abdominal sepsis, and short bowel syndrome in cases of extensive resection [3,6]. Close postoperative monitoring is therefore essential, particularly in frail elderly patients [6].

While midgut volvulus is often described in infants and occasionally in younger adults, this case highlights that primary volvulus can also occur in the 10th decade of life [2,5]. Recent case reports describe similar presentations in elderly and even nonagenarian patients, most often with secondary causes, making this instance of primary volvulus especially rare [5]. The successful outcome in this patient underscores that timely surgical management with appropriate perioperative support can achieve favorable results, even in the very elderly [1,3].

## Conclusions

PSIV is an uncommon clinical entity, particularly in the very elderly, and often presents with vague or muted symptoms that can delay diagnosis. This case emphasizes the need to consider PSIV in the differential diagnosis of acute abdominal pain in older adults, especially when imaging demonstrates features such as the whirlpool sign. Although operative risks are higher in this population, prompt surgical exploration remains essential, as delays can lead to irreversible ischemia, necrosis, and significant mortality.

Our patient’s successful outcome demonstrates that, with timely recognition and appropriate operative management, even high-risk elderly individuals can recover well. Continued clinical awareness and early intervention are crucial for improving outcomes in this rare but potentially life-threatening condition.
